# Supplementary material for: Method for cycle detection in sparse, irregularly sampled, long-term neuro-behavioral timeseries: Basis pursuit denoising with polynomial detrending of long-term, inter-ictal epileptiform activity
Source: PLoS Comput Biol. 2024 Apr 25;20(4):e1011152. doi: 10.1371/journal.pcbi.1011152 (PMC11045138; doi:10.1371/journal.pcbi.1011152)
Supplement: S1 Supplementary Materials — (DOCX) [file pcbi.1011152.s001.docx]

**Supplementary materials**

**Supplementary methods**

*Reducing the 2D minimization problem to 1D via variable projection:* The initial expression for basis pursuit with polynomial detrending (BPWP) (Equation 1) is minimized with respect to both *x* and *z*. As described in the materials and methods in more detail, A is the product of binary row subsampling matrix Φ and discrete cosine transform (DCT) basis Ψ, B is the product of binary row subsampling matrix Φ and Vandermonde polynomial basis Τ, *y* is the vector of samples, *z* is the vector of polynomial coefficients, *x* is the vector of DCT coefficients, and the parameter δ captures error and constrains the L1 norm of the DCT coefficient vector.

| $\min_{x,z} \left\Vert y-Ax-Bz \right\Vert_{2}^{2} s.t. \left\Vert x \right\Vert_{1} \leq\delta$ | (1) |
| --- | --- |

We used variable projection{Golub, 1973 #727}{Golub, 2003 #728} to simplify the expression to a 1D minimization problem (Equation 2). The model’s solution for *x* is then input into equation 3 to solve for *z*.

| $\min_{x} \left\Vert(I-BB^{+})(y-Ax) \right\Vert_{2}^{2} s.t. \left\Vert x \right\Vert_{1} \leq\delta$ | (2) |
| --- | --- |
| $\hat{\boldsymbol{z}}=\boldsymbol{B}^{+}(\boldsymbol{y}-\boldsymbol{Ax})$ | (3) |

The associated derivation is shown here:

Given $\left\| y-Ax-Bz \right\|_{2}^{2} we differentiate with respect to z, set the residual to 0, and solve:$

$\frac{\partial\left\| \boldsymbol{y}-\boldsymbol{Ax}-\boldsymbol{Bz} \right\|_{2}^{2}}{\partial\boldsymbol{z}}=0$

$\frac{\partial(\boldsymbol{y}^{'}\boldsymbol{y}-2\boldsymbol{y}^{'}\boldsymbol{Ax}-2{\boldsymbol{z}^{'}\boldsymbol{B}}^{'}\boldsymbol{y}+2{\boldsymbol{z}^{'}\boldsymbol{B}}^{'}\boldsymbol{Ax}+{\boldsymbol{z}^{'}\boldsymbol{B}}^{'}\boldsymbol{Bz})}{\partial\boldsymbol{z}}=0$

$$-2\boldsymbol{B}^{'}\boldsymbol{y}+2\boldsymbol{B}^{'}\boldsymbol{Ax}+{2\boldsymbol{B}}^{'}\boldsymbol{Bz}=0$$

$$\boldsymbol{B}^{\boldsymbol{'}}\boldsymbol{Bz}=\boldsymbol{B}^{\boldsymbol{'}}\boldsymbol{y}-2\boldsymbol{B}^{\boldsymbol{'}}\boldsymbol{Ax}=\boldsymbol{B}'(\boldsymbol{y}-\boldsymbol{Ax})$$

$$\hat{\boldsymbol{z}}={\left( \boldsymbol{B}^{\boldsymbol{'}}\boldsymbol{B} \right)^{-1}\boldsymbol{B}}^{'}(\boldsymbol{y}-\boldsymbol{Ax})$$

$\hat{\boldsymbol{z}}=\boldsymbol{B}^{+}(\boldsymbol{y}-\boldsymbol{Ax})$ $B^{+}=pseudoinverse$*, via Moore-Penrose inverse*

We then replace z in the original objective function with the variable projection-derived estimate:

$$\left\| y-Ax-Bz \right\|_{2}^{2}$$

$$\left\| \left( y-Ax \right)-BB^{+}\left( y-Ax \right) \right\|_{2}^{2}$$

$$\left\| \left( I-BB^{+} \right)(y-Ax) \right\|_{2}^{2}$$

This yields the 1d constrained optimization problem:

$\min_{x} \left\| (I-BB^{+})(y-Ax) \right\|_{2}^{2} s.t. \left\| x \right\|_{1} \leq\delta$

*Customized frequency representation in the DCT basis:* To minimize the disruption of the orthonormal basis structure, we made targeted modifications to the default frequency representation for the DCT. Briefly, to define *f(k),* we increased the density of frequency representation within the range of interest on the low frequency end at the expense of randomly removing an equivalent number of frequencies from the high frequency end.

| Defining *f(k)*: |  |  |
| --- | --- | --- |
| 1. Begin with default frequency representation: $f=fs*\frac{\left( 0:N-1 \right)}{2N}$ | Length N |  |
| 1. Define frequency range of interest (Hz): $f_{min}=p, f_{max}=q$ |  |  |
| 1. Define density factor: $p$ |  |  |
| 1. Define high density vector: $linspace(f_{min}, f_{max}, p)$ | Length p |  |
| 1. Insert high density vector into default frequency representation | Length N+p |  |
| 1. Randomly remove $p$ frequencies in the high frequency range. | Length N |  |

These changes in frequency representation are designed to improve representation of frequencies of interest while attempting to preserve as much orthogonality of the basis as possible. Given the non-uniform spacing of frequencies in the basis, one might be tempted to include a post-hoc weighting vector to accommodate the non-uniformity in the frequencies. Introducing a weighting vector would alter the error approximation and potentially cause noise amplitication. To avoid the introduction of noise or bias, any weighting vector would have to be carried throughout out the regression expression, i.e. captured in the regression expression describing the relationship between *y*, *Ax*, and $\delta$. Adding a vector of weights as such would change the expression from least squares to weighted least squares, which has a different, frequency-specific representation of noise variance. In our current formulation, we maintain the assumption that noise is iid over time.

*Features and parameters:* Several model components can be adjusted depending on the application.

- $\boldsymbol{\delta}$**:** $\delta$ describes the measurement and model error. This composite error term should be selected based on parameter sweeps and cross validation to identify the value of $\delta$ which minimizes a quality metric such as mean square error.
- $\boldsymbol{dt}$**:** Although the samples in *y* are collected irregularly, with irregular inter-sample intervals, the samples in y must be described according to relative differences in continuous time in the subsampling matrix. The unit of time *dt* dictates the density of representation of real time and consequently both how sparse the input data are (for a fixed length of y, a smaller dt means sparser input data), and *n,* the length of the full array and consequently the dimensions of the DCT basis.
- $\boldsymbol{n:}$ The total length of the full array, *n,* is a consequence of the choice of *dt* and dictates the number of DCT points.
- $\boldsymbol{\Psi}$**:** $\Psi$ is the spectral, DCT basis. The frequencies represented in the DCT basis depend in part on *n*.
- $\mathbf{p,T}$**:** $T$ is the polynomial basis Vandermonde matrix. The largest degree captured in the polynomial basis is *p*-1. The maximal polynomial degree should align with anticipated or known trends in the data, most appropriately low-order polynomials.

*Assumptions:* Key assumptions of BPWP and similar methods are that the component oscillations are narrowband, or separable, and that the signal being reconstructed in sparse{Puy, 2011 #716}. Randomness is a sufficient condition that can improve recoverability. Data must be irregularly sampled because samples collected at regular intervals introduce false cycles at the interval duration.

*Reconstructing the underlying continuous timeseries:* The DCT and polynomial coefficient outputs can be used to reconstruct the underlying timeseries. The estimated timeseries $\hat{y}$ (equation 12) is the sum of the inverse DCT transform (equation 10) of the DCT coefficients $\theta$ and the polynomial timeseries $\rho$ (equation 11) based on the polynomial coefficients.

| $\theta_{n}= \sqrt{\frac{2}{N}}\sum_{n=1}^{N} x(k)\frac{1}{\sqrt{1+\delta_{k1}}}\cos(\frac{\pi}{2N}(2n-1)(f(k)-1))$ | $n=0:N-1$ | (10) |
| --- | --- | --- |
| $\rho= \sum_{i=0}^{\max degree} z_{i}t^{i}$ | $t=t:N$ | (11) |
| $\hat{y}= \theta+ \rho$ |  | (12) |

*Parameter sweeps:* We used patient-specific, 10-fold 75/25 cross validation to identify an appropriate value for $\delta$. Time domain mean squared error (MSE) (Equation 13) evaluating the difference between the input data y and the output data $\hat{y}$ was the quality metric.

| ${MSE}_{time}=\frac{1}{n}\sum_{i=1}^{n} {(y_{i}-\hat{y}_{i})}^{2}$ | (13) |
| --- | --- |

For a range of $\delta$ values we randomly removed 25 percent of observations from the input data and used MSE to compare the removed observations with their values estimated by the model. Rates and cycles of epileptiform activity are highly patient-specific, necessitating an individualized approach to parameter selection. Results from 90/10 cross validation are available in the supplementary materials.

*Simulated data for cycle detection under high and low SNR conditions:* The amplitude of the underlying oscillation was varied to depict high and low variance signals. The relative amplitudes of the underlying oscillation and added gaussian noise were varied to depict cases with high and low SNR. The simulated timeseries were randomly sampled at frequencies ranging from a few samples per week to a few samples per day.
