# Supplementary figures and images for: Method for cycle detection in sparse, irregularly sampled, long-term neuro-behavioral timeseries: Basis pursuit denoising with polynomial detrending of long-term, inter-ictal epileptiform activity
Source: PLoS Comput Biol. 2024 Apr 25;20(4):e1011152. doi: 10.1371/journal.pcbi.1011152 (PMC11045138; doi:10.1371/journal.pcbi.1011152)

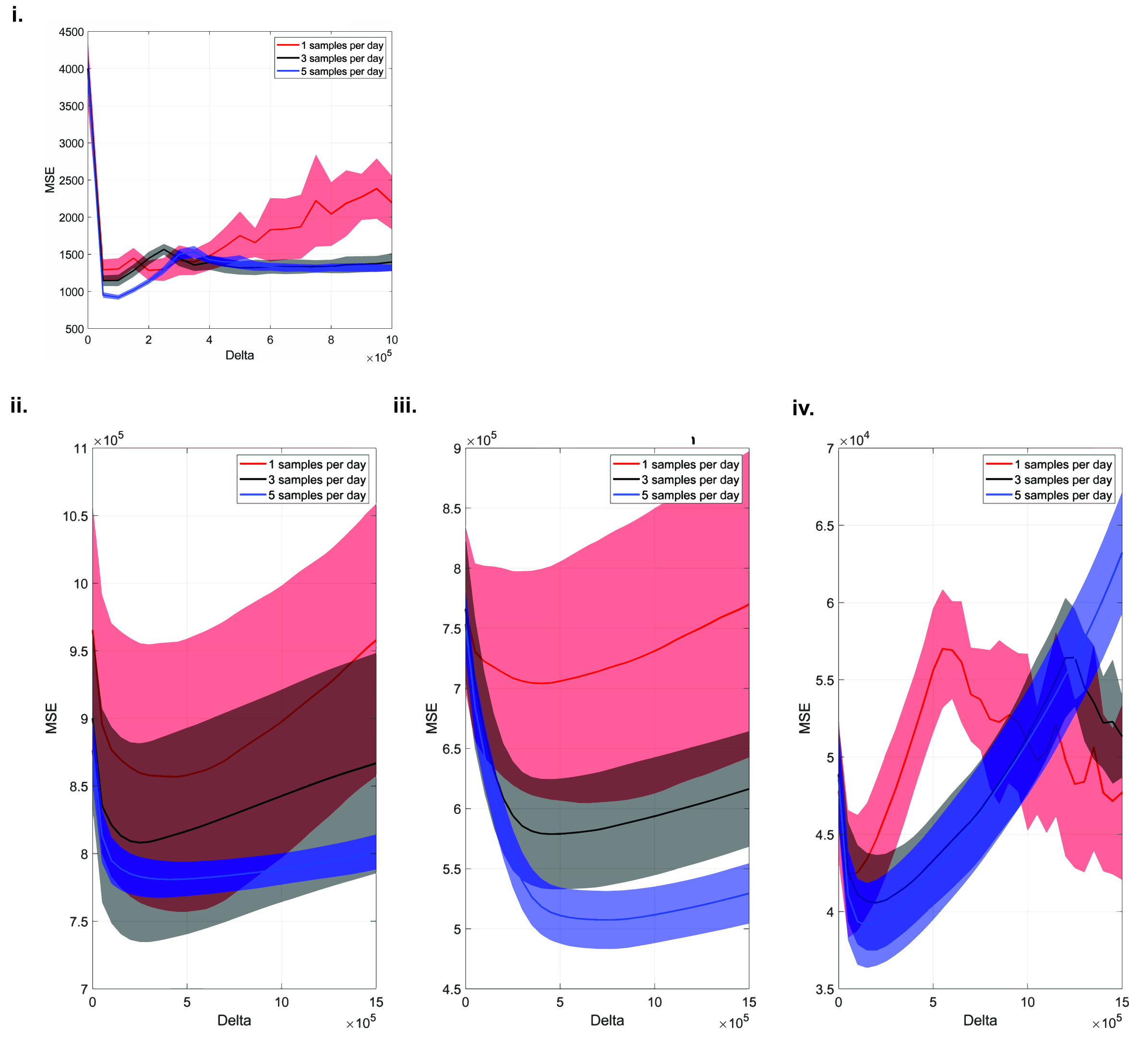

Supplement: S1 Fig — Mean square error (MSE) between the real and estimated samples was calculated for each iteration of δ parameter and density of random sampling. (i) Shows results from the simulated IED timeseries. Subplots (ii), (iii), (iv) show results from real-world IED timeseries from participants 3, 1, and 2 respectively. (TIF) [file pcbi.1011152.s002.tif]

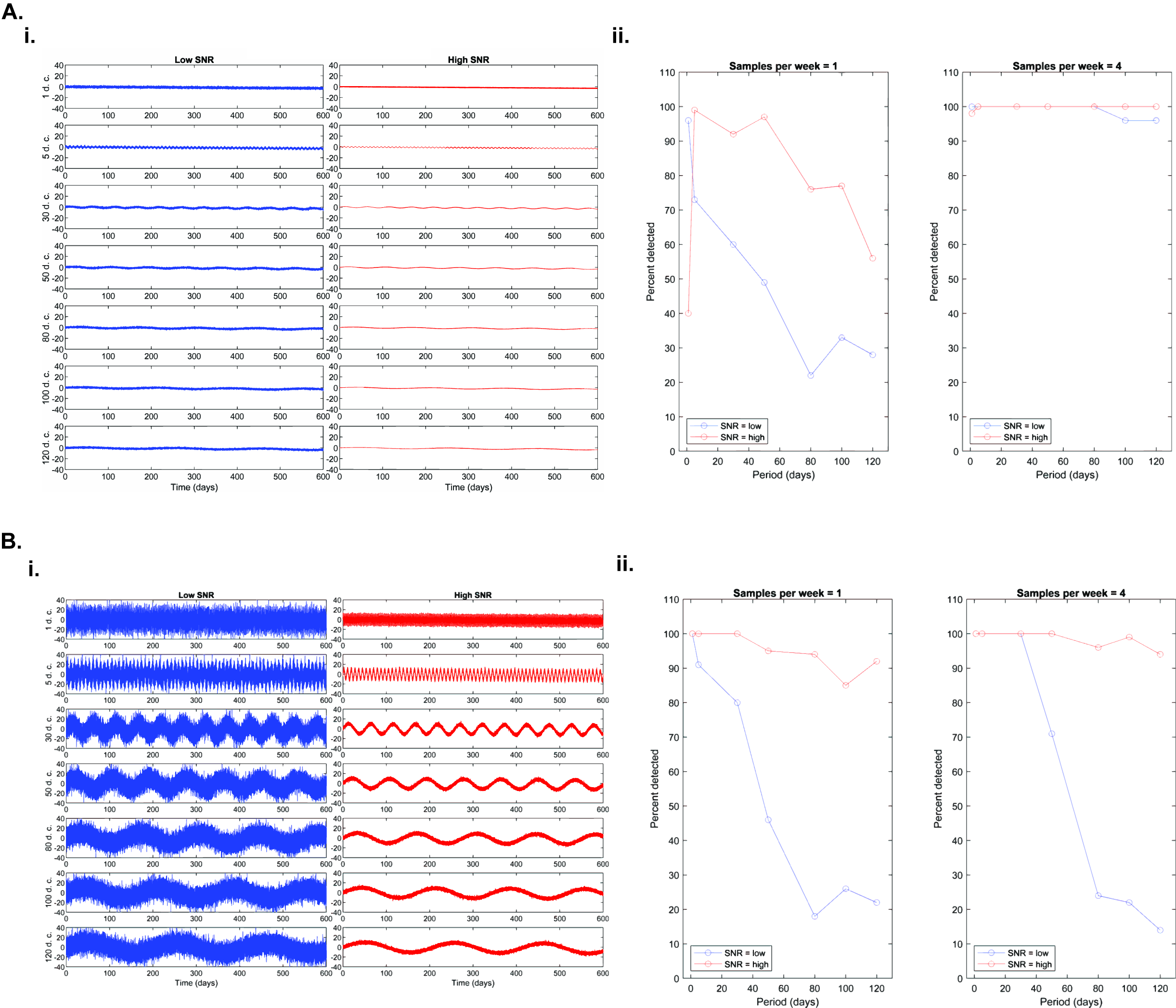

Supplement: S2 Fig — Signals of different cycle length, variance, and SNR were generated and input to BPWP. (A) Low variance condition. (i) Example signals with low and high SNR are shown in blue and red. Y axis labels with “d. c.” denotes “day cycle”, capturing the period for each simulated oscillation. (ii) Simulated signals were resampled and BPWP was recalculated 10 times. The percent of iterations wherein the true oscillation was captured as a significant peak constitutes the percent detected metric on the y axis. Detection rate is plotted for a low sampling (1 random sample per week) and higher sampling (4 random samples per week) condition. Part (B) mirrors part (A), but for simulated signals in the high variance condition. (TIF) [file pcbi.1011152.s003.tif]

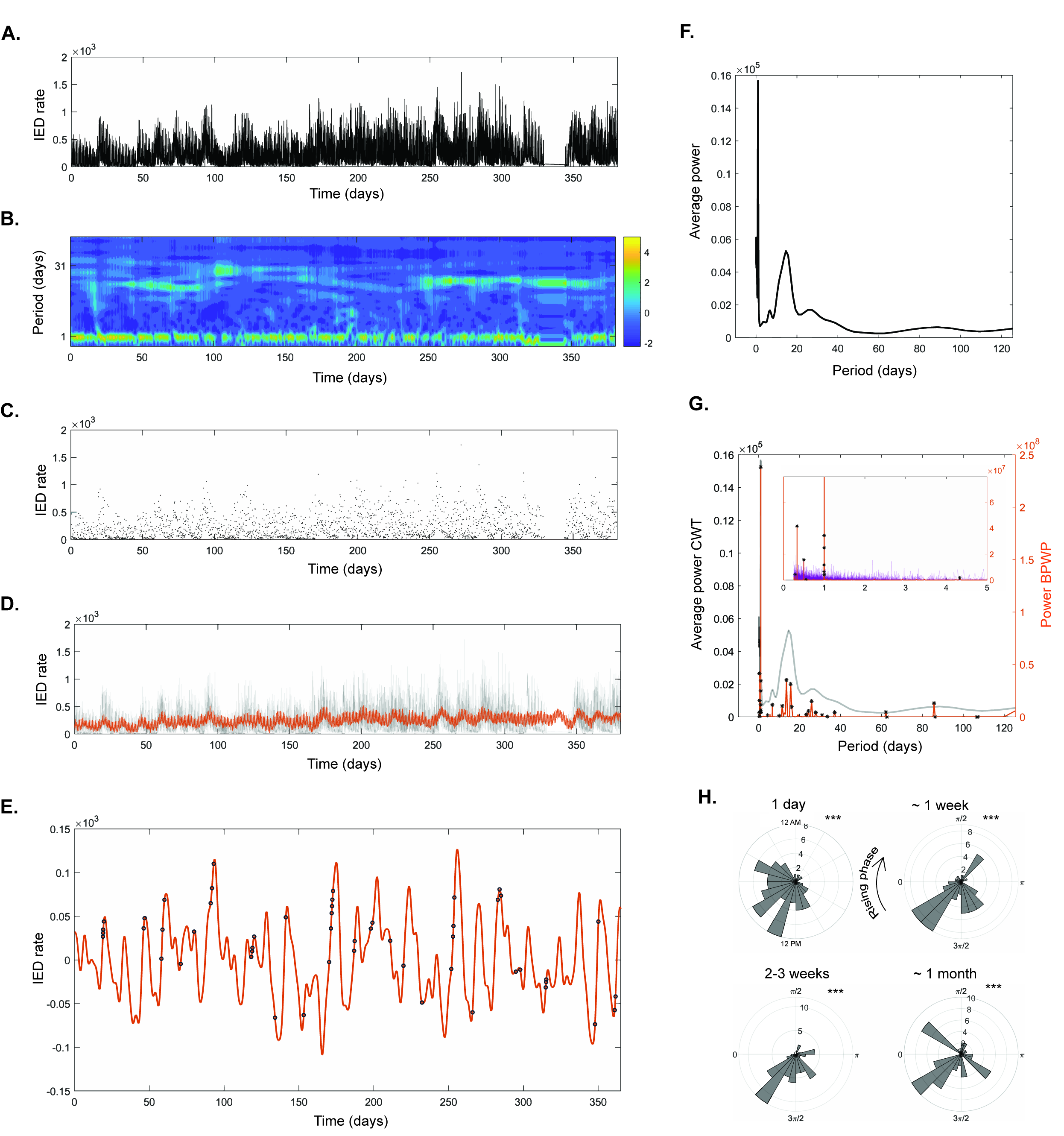

Supplement: S3 Fig — (A) Raw data showing hourly rate of IEDs detected from the left hippocampus, updated every 20 minutes. Timeseries consists of over 20,000 samples. (B) Complex wavelet transform (CWT) spectrogram of the timeseries in A showing power in different cycles. Strong cycles are evident at one day and around on month. (C) Random samples from the raw data in A averaging at 5 samples per day. Timeseries consists of 1,800 total samples. (D) Underlying raw data are shown in gray. BPWP’s estimated reconstruction of the underlying data based on the sparse samples in (C) is shown in orange. (E) The method’s estimated reconstruction of the underlying signal using only significant peaks from (G) with a period longer than two days is in orange. Overlayed black circles denote when seizures occurred. Seizures appear to prefer the peaks of the combined slow cycles derived from the method. (F) Average of CWT spectrum (averaged over time from the spectrogram in (B) shows cycles of IED rate including periods around one day, two-three weeks, one month, and 90 days. (G) The CWT spectrum for the raw data is shown in gray. The method’s spectral output is shown in orange. Black stars denote significant peaks; peaks whose amplitude was above the 99th percentile of the distribution created by shuffling the input data and re-calculating the method 100 times. The insert shows the spectral outputs from the reshuffling in purple. (H) The BPWP-based reconstruction was filtered in cycle ranges around one day, one week, two to three weeks, and one month then Hilbert transformed to identify the phase at which seizures occurred for each of these cycles. Polar histograms denoting the phase at which seizures occurred for each of these cycles indicate a cycle-specific phase preference for seizures. Stars denote p < 0.001 on the Omnibus test for uniformity, indicating that seizure phase is not uniformly distributed. (TIF) [file pcbi.1011152.s004.tif]

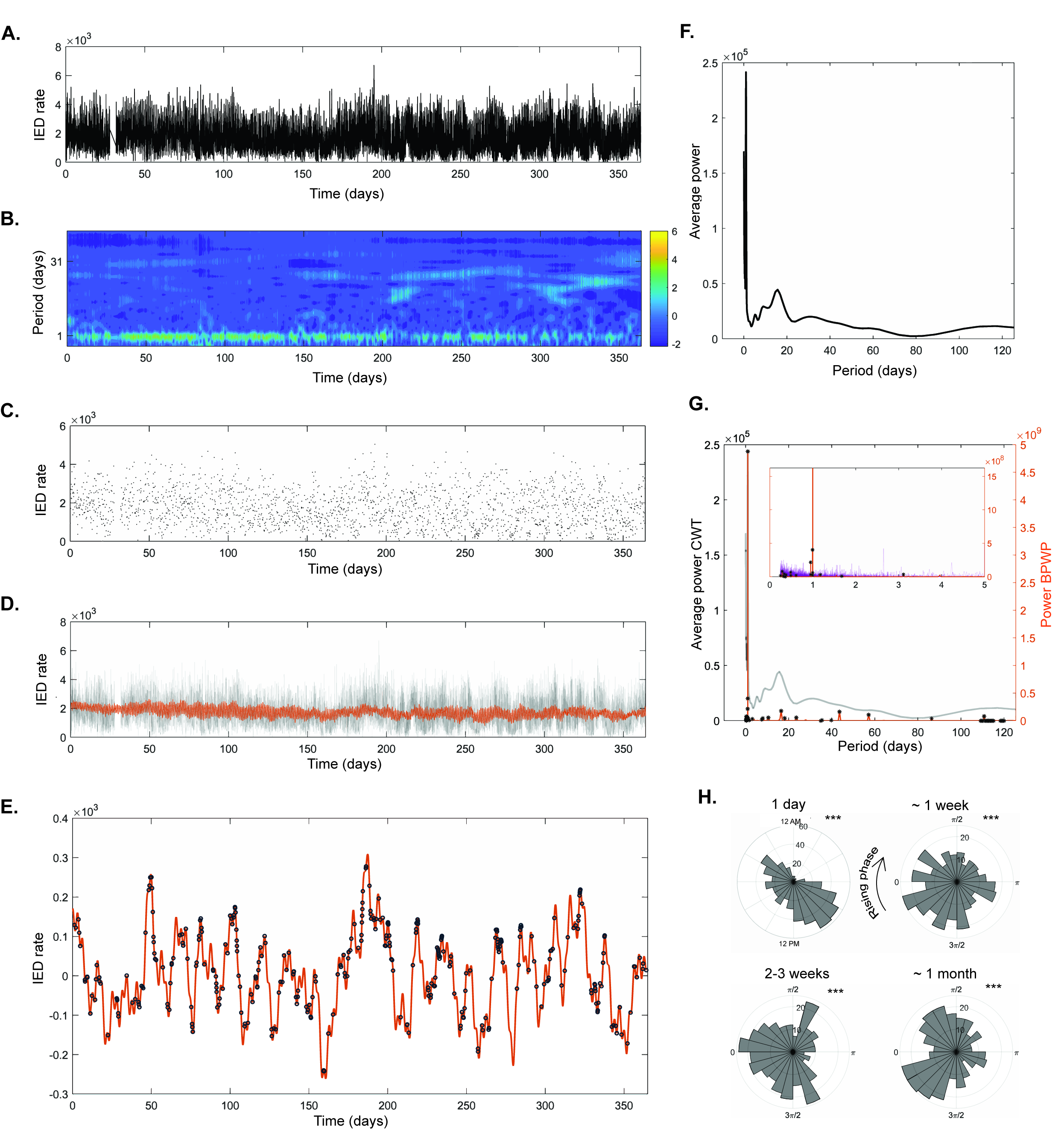

Supplement: S4 Fig — (A) Raw data showing hourly rate of IEDs detected from the left hippocampus, updated every 20 minutes. Timeseries consists of over 20,000 samples. (B) Complex wavelet transform (CWT) spectrogram of the timeseries in A showing power in different cycles. Strong cycles are evident at one day and around on month. (C) Random samples from the raw data in (A) averaging at 5 samples per day. Timeseries consists of 1,800 total samples. (D) Underlying raw data are shown in gray. The estimated reconstruction of the underlying data based on the sparse samples in (C) is shown in orange. (E) The method’s estimated reconstruction of the underlying signal using only significant peaks from F with a period longer than two days is in orange. Overlayed black circles denote when seizures occurred. (F) Average of CWT spectrum (averaged over time from the spectrogram in B) shows cycles of IED rate including periods around one day, two weeks, and one month. (G) The CWT spectrum for the raw data is shown in gray. The method’s spectral output is shown in orange. Black stars denote significant peaks; peaks whose amplitude was above the 99th percentile of the distribution created by shuffling the input data and re-calculating the method 100 times. The insert shows the spectral outputs from the reshuffling in purple. (H) The method-based reconstruction was filtered in cycle ranges around one day, one week, two to three weeks, and one month then Hilbert transformed to identify the phase at which seizures occurred for each of these cycles. Polar histograms denoting the phase at which seizures occurred for each of these cycles indicate a cycle-specific phase preference for seizures. Stars denote p < 0.001 on the Omnibus test for uniformity, indicating that seizure phase is not uniformly distributed. (TIF) [file pcbi.1011152.s005.tif]

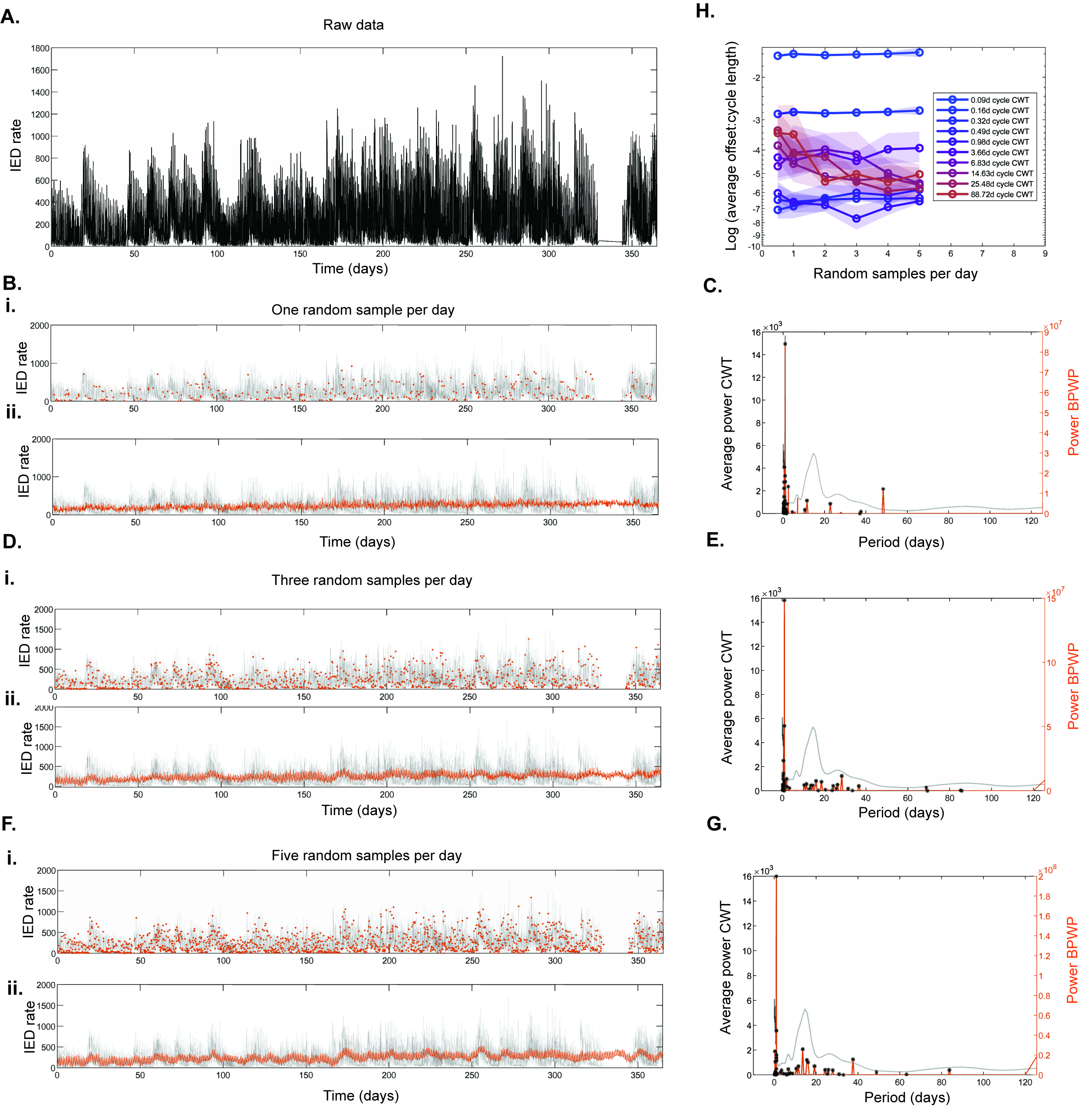

Supplement: S5 Fig — (A) Raw data showing hourly rate of IEDs detected from the left hippocampus, updated every 20 minutes. Timeseries consists of over 20,000 samples. (Bi) Raw data are in gray and one random sample per day are in orange. (Bii) Raw data are in gray and the reconstructed signal using model outputs based on input data of one random sample per day is in orange. (C) Average complex wavelet transform (CWT) spectrum from the raw data in (A) is in gray. BPWP spectral output based on one sample per day input is shown in orange. Black stars denote significant peaks; peaks whose amplitude was above the 99th percentile of the distribution created by shuffling the input data and re-calculating BPWP 100 times. Random samples, signal reconstructions, and BPWP spectra are shown again for sampling rates of three and five per day in (D) and (E) and in (F) and (G) respectively. Agreement between BPWP output and raw data and CWT spectra improves as the signal is sampled more densely. Part (H) shows agreement between the BPWP and CWT spectra as a function of frequency of random sampling. For each sampling frequency, the raw data were re-sampled and BPWP was re calculated 10 times. For each peak in the CWT spectrum, the offset between the period of the CWT peak and the nearest BPWP peak was calculated in terms of days and divided by the period of the CWT peak. This offset-to-cycle-length ratio was averaged across the 10 iterations and plotted as a log value on the y axis. The associated frequency of random sampling was plotted on the x axis. Shaded areas denote 95% confidence intervals. The offset ratio decreases and stabilizes as sampling density increases. (TIF) [file pcbi.1011152.s006.tif]

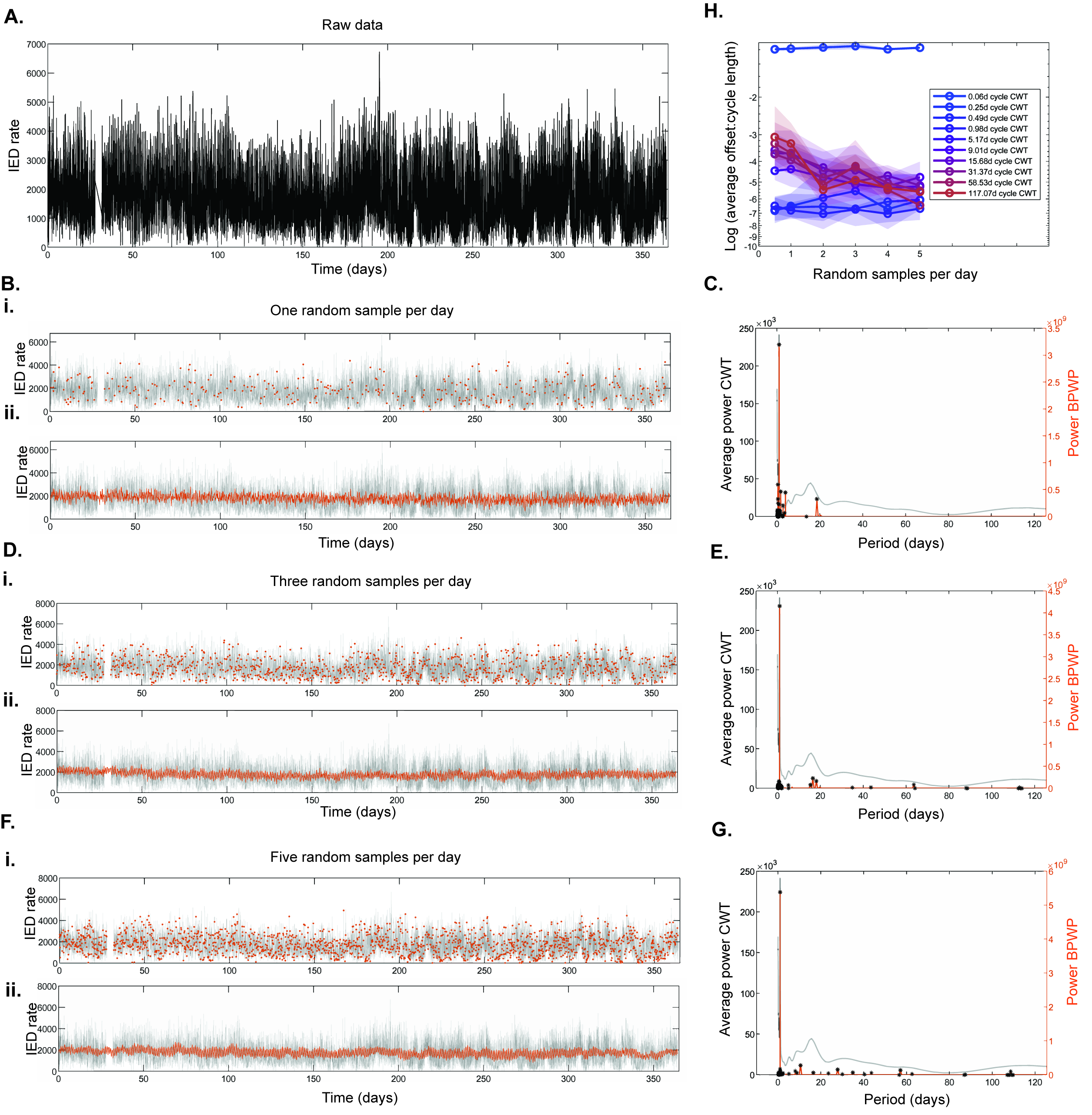

Supplement: S6 Fig — (A) Raw data showing hourly rate of IEDs detected from the left hippocampus, updated every 20 minutes. Timeseries consists of over 20,000 samples. (Bi) Raw data are in gray and one random sample per day are in orange. (Bii) Raw data are in gray and the reconstructed signal using model outputs based on input data of one random sample per day is in orange. (C) Average complex wavelet transform (CWT) spectrum from the raw data in (A) is in gray. The BPWP spectral output based on one sample per day input is shown in orange. Black stars denote significant peaks; peaks whose amplitude was above the 99th percentile of the distribution created by shuffling the input data and re-calculating BPWP 100 times. Random samples, signal reconstructions, and BPWP spectra are shown again for sampling rates of three and five per day in (D) and (E) and in (F) and (G) respectively. Agreement between BPWP output and raw data and CWT spectra improves as the signal is sampled more densely. Part (H) shows agreement between the BPWP and CWT spectra as a function of frequency of random sampling. For each sampling frequency, the raw data were resampled and the BPWP was re calculated 10 times. For each peak in the CWT spectrum, the offset between the period of the CWT peak and the nearest BPWP peak was calculated in terms of days and divided by the period of the CWT peak. This offset-to-cycle-length ratio was averaged across the 10 iterations and plotted as a log value on the y axis. The associated frequency of random sampling was plotted on the x axis. Shaded areas denote 95% confidence intervals. (TIF) [file pcbi.1011152.s007.tif]

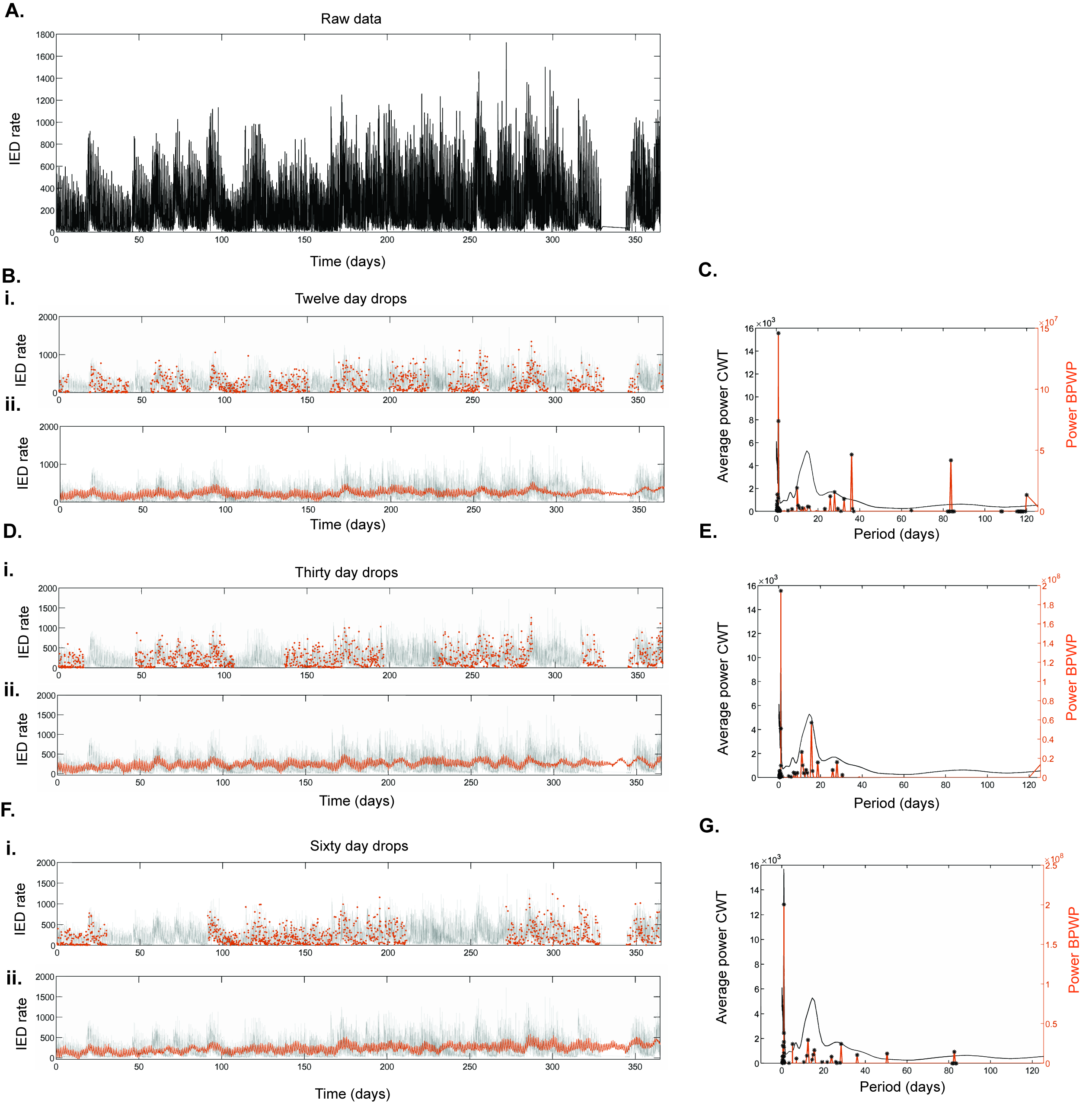

Supplement: S7 Fig — (A) Raw data showing hourly rate of IEDs detected from the left hippocampus, updated every 20 minutes. Timeseries consists of over 20,000 samples. (Bi) Raw data are in gray and random sampling excluding 12-day data drops are in orange. (Bii) Raw data are in gray and the reconstructed signal using model output based on input data with 12-day data drops is in orange. C) Average complex wavelet transform (CWT) spectrum from the raw data in (A) is in gray. The BPWP spectral output based on the sampling in (Bi) input is shown in orange. Black stars denote significant peaks; peaks whose amplitude was above the 99th percentile of the distribution created by shuffling the input data and re-calculating BPWP 100 times. Data drops of thirty- and sixty- days duration, signal reconstructions, and method spectra are shown in (D) and (E) and in (F) and (G) respectively. The total number of samples for BPWP is fixed across the conditions at n = 1307 which is approximately 4 samples per day assuming no drops. (TIF) [file pcbi.1011152.s008.tif]

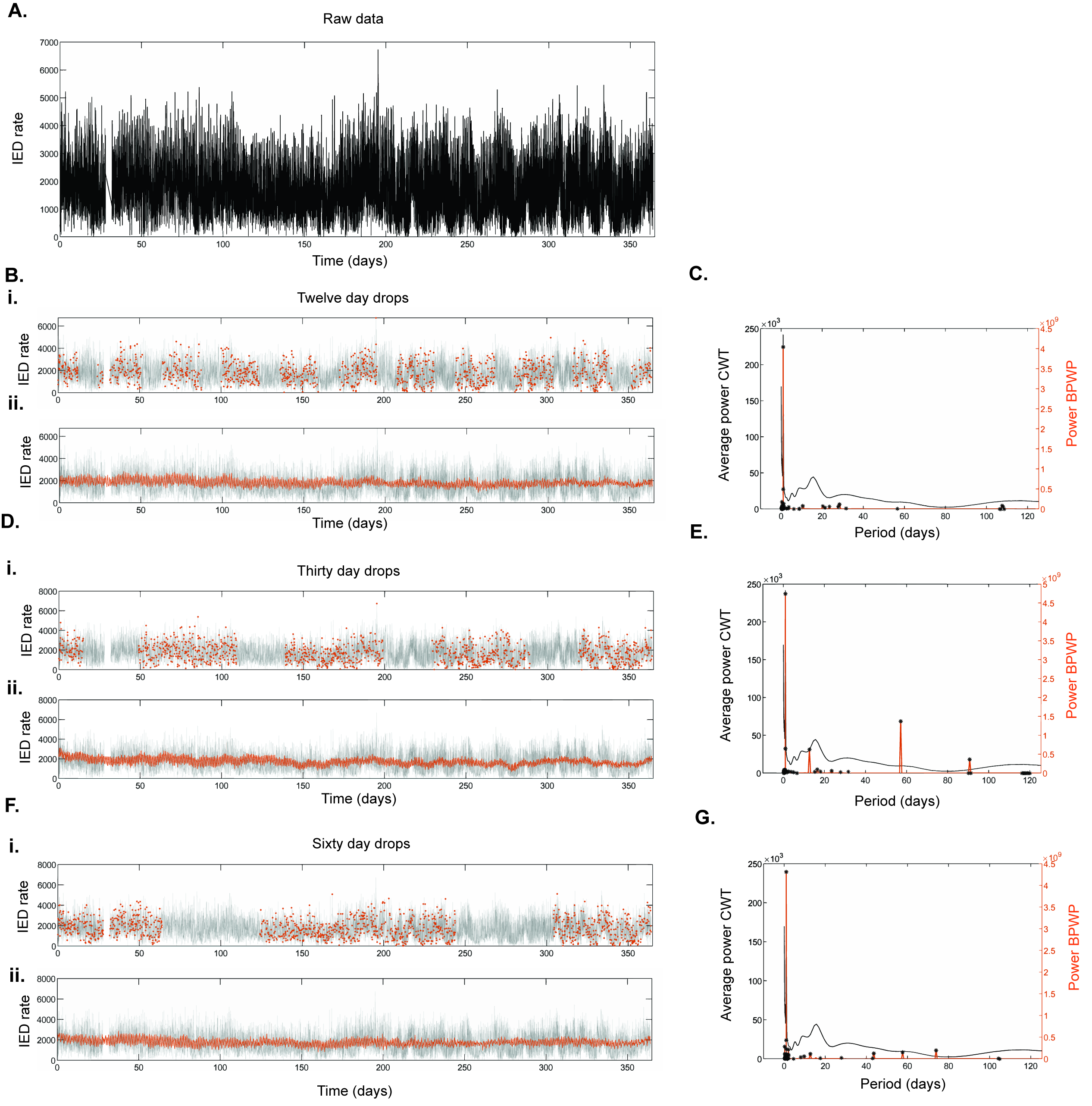

Supplement: S8 Fig — (A) Raw data showing hourly rate of IEDs detected from the left hippocampus, updated every 20 minutes. Timeseries consists of over 20,000 samples. (Bi) Raw data are in gray and random sampling excluding 12-day data drops are in orange. (Bii) Raw data are in gray and the reconstructed signal using model output based on input data with 12-day data drops is in orange. (C) Average complex wavelet transform (CWT) spectrum from the raw data in (A) is in gray. The BPWP spectral output based on the sampling in (Bi) input is shown in orange. Black stars denote significant peaks; peaks whose amplitude was above the 99th percentile of the distribution created by shuffling the input data and re-calculating BPWP 100 times. Data drops of thirty- and sixty- days duration, signal reconstructions, and method spectra are shown in (D) and (E) and in (F) and (G) respectively. The total number of samples for BPWP is fixed across the conditions at n = 1307 which is approximately 4 samples per day assuming no drops. (TIF) [file pcbi.1011152.s009.tif]

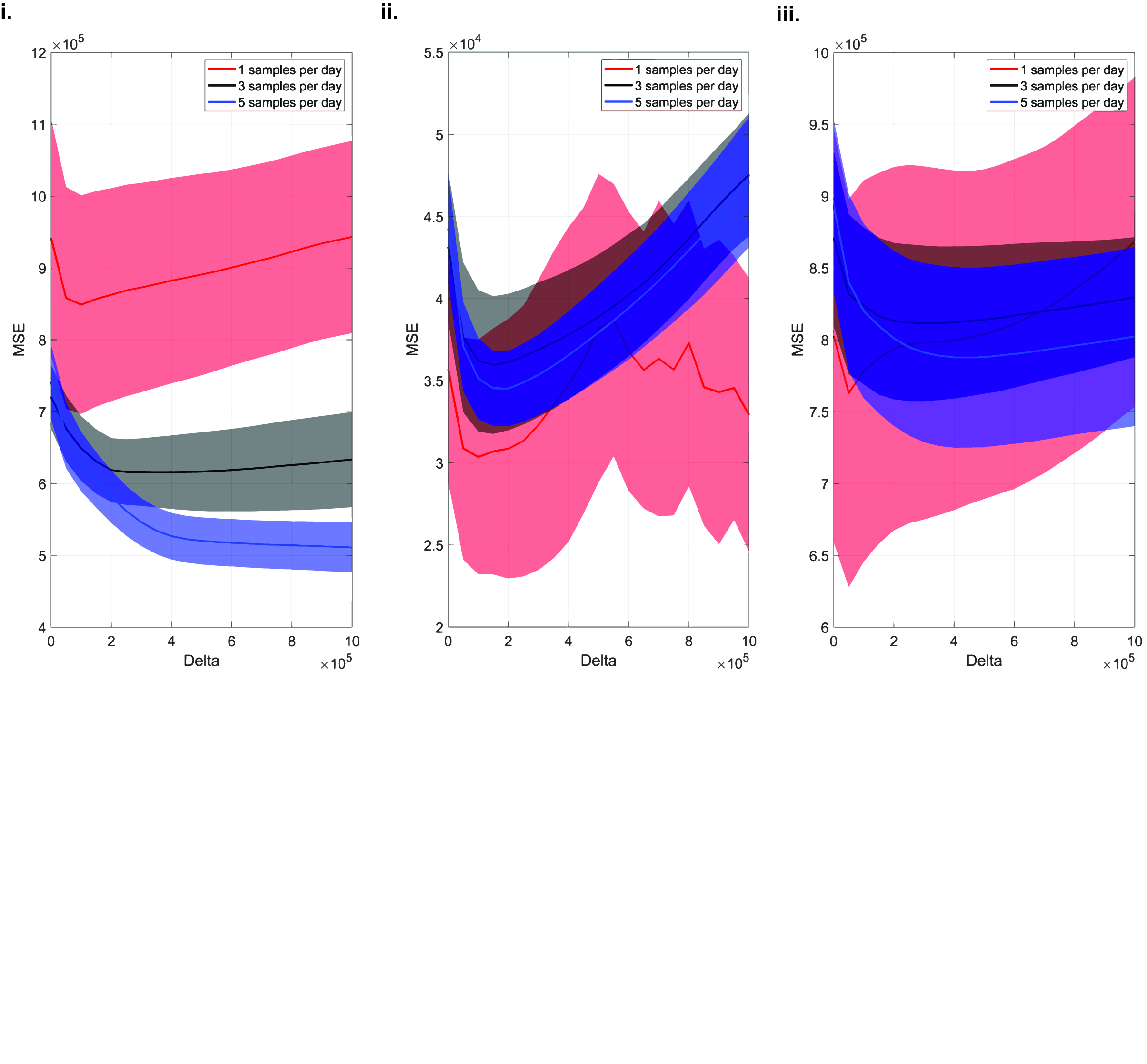

Supplement: S9 Fig — Mean square error (MSE) between the real and estimated samples was calculated for each iteration of δ parameter and density of random sampling. Subplots (i), (ii), (iii) show results from real-world IED timeseries from participants 1, 2, and 3 respectively. (TIF) [file pcbi.1011152.s010.tif]

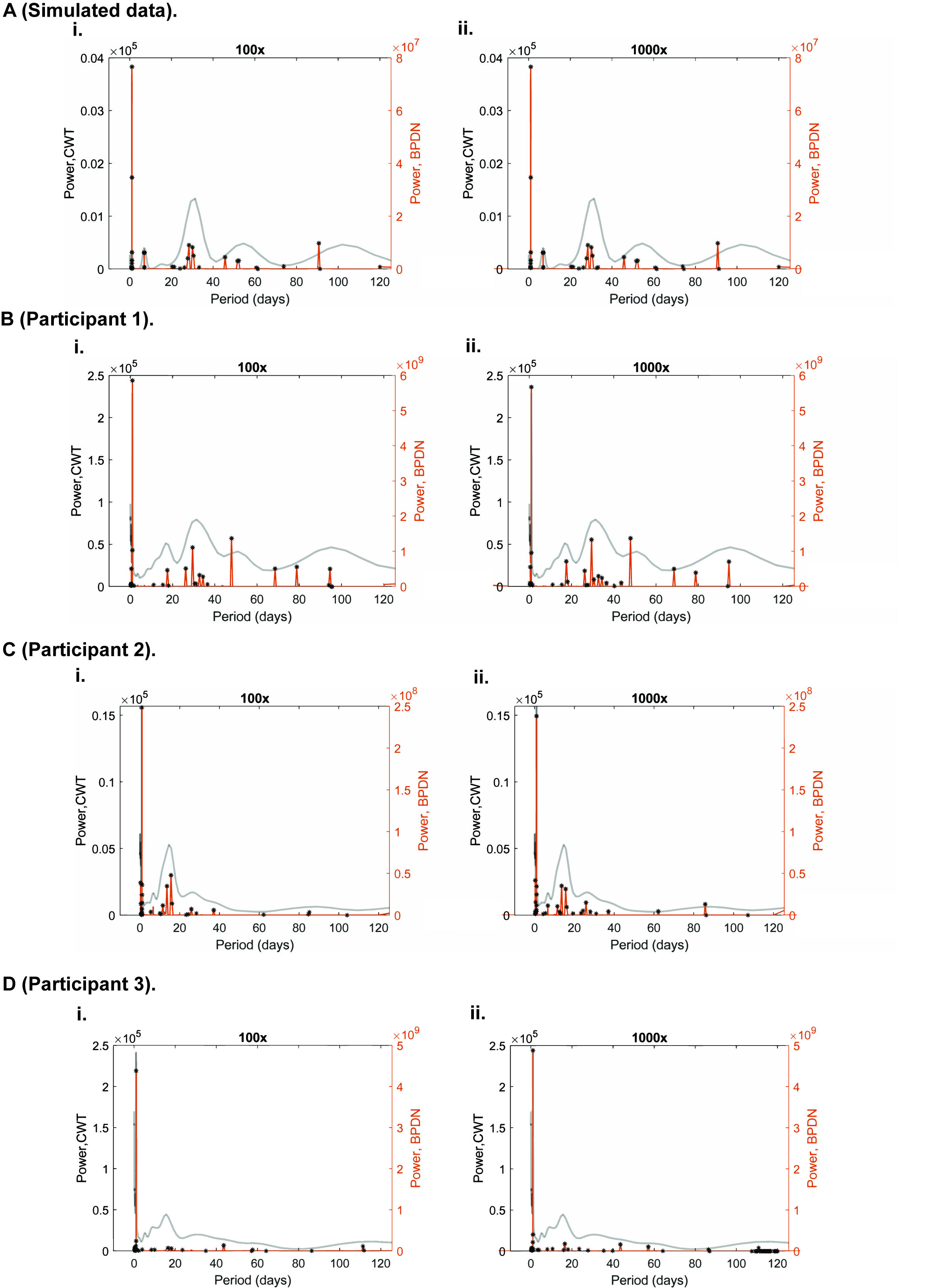

Supplement: S12 Fig — Significant peaks in the output power spectra from the simulated data, participants 1, 2, and 3 are shown in sections (A), (B), (C), and (D) respectively. In i, the reference distribution for significance calculations was the result of re-running the model with re-shuffled input data 100 times. In ii, the model was re-run 1,000 times. (TIF) [file pcbi.1011152.s013.tif]

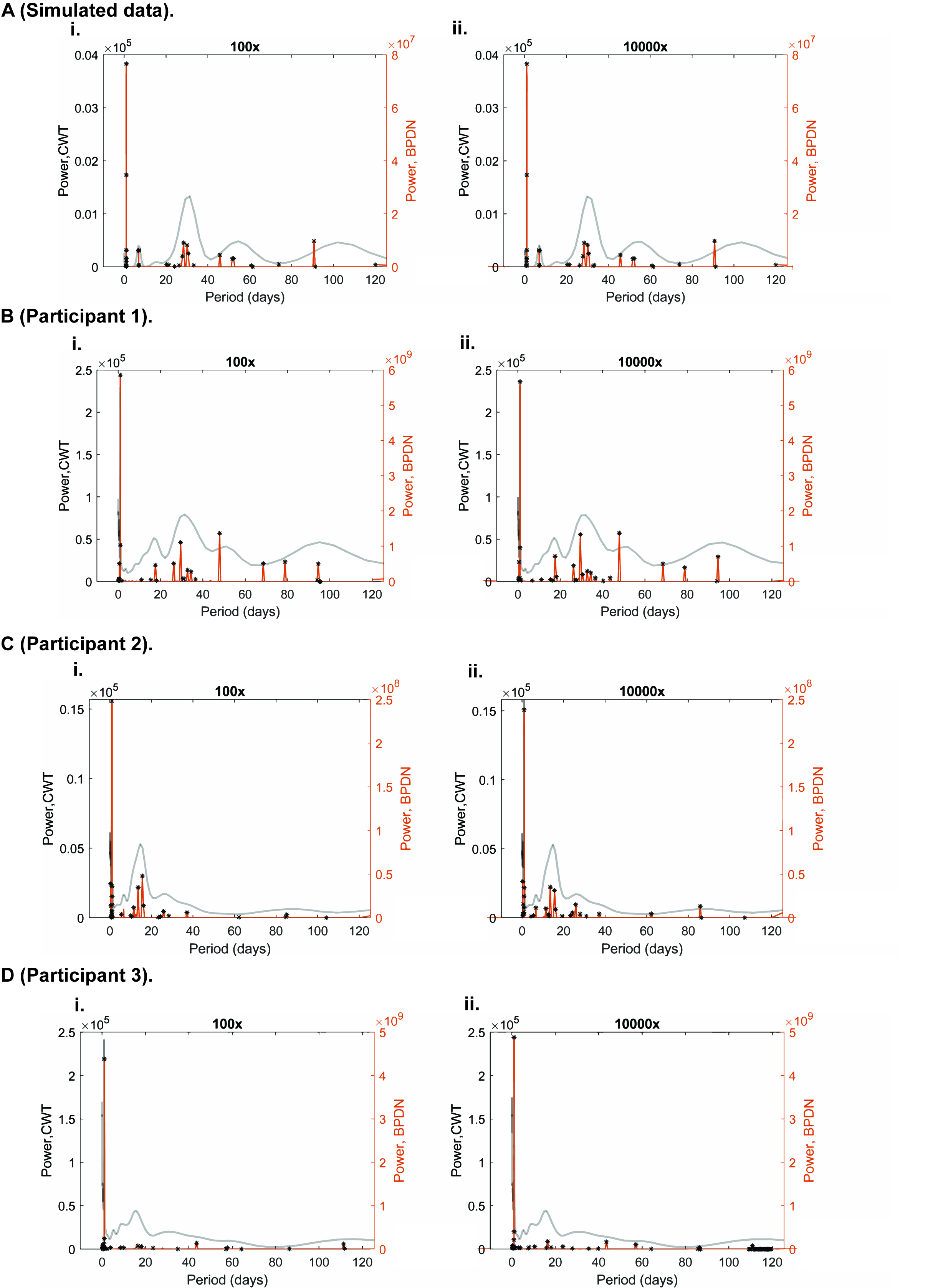

Supplement: S13 Fig — Significant peaks in the output power spectra from the simulated data, participants 1, 2, and 3 are shown in sections (A), (B), (C), and (D) respectively. In i, the reference distribution for significance calculations was the result of re-running the model with re-shuffled input data 100 times. In ii, the model was re-run 10,000 times. (TIF) [file pcbi.1011152.s014.tif]
