# Supplementary material for: Method for cycle detection in sparse, irregularly sampled, long-term neuro-behavioral timeseries: Basis pursuit denoising with polynomial detrending of long-term, inter-ictal epileptiform activity
Source: PLoS Comput Biol. 2024 Apr 25;20(4):e1011152. doi: 10.1371/journal.pcbi.1011152 (PMC11045138; doi:10.1371/journal.pcbi.1011152)
Supplement: Attachment — Submitted filename: ResponseToReviewers.docx [file pcbi.1011152.s015.docx]

**Response to Reviewers**

***Thank you to the Reviewers for their close reading and consideration of this manuscript.***

Reviewer 1:

Minor concern:
- recomputing 100 times and taking the 99th percentile does not make much sense to me. I would recommend recomputing 10000 or better yet 100000 times and using the 99th percentile. This leaves much less to randomness and gets a more clear boundary of the distribution. Unless it is the case that only 100 shufflings are sufficient to fully sample the distribution, which I suspect is not the case.

***To evaluate the stability of the significance testing we repeated the methodology by recomputing BPWP on reshuffled data for 1,000 and 10,000 iterations. The spectral outputs for each approach are shown in Supplementary figures 12 and 13 together with the outputs from the 100-iteration case. By visual inspection, the spectra are sufficiently similar between the 100, 1,000, and 10,000 iteration cases. Additional text referencing these figures was added to the methods section in the main manuscript (Lines 465-468) and discussion (Line 348).***

Reviewer 2:

1. The authors mention that the BPWP algorithm is computationally intensive and not suitable for real-time analysis on an embedded system. It would be helpful to include additional information on how the computational time scales with the length of data. This would provide insights into the scalability of the approach and potential limitations when dealing with larger datasets.

***We included additional descriptions of runtimes from our implementation. Building the non-parametric, reference distribution to describe the statistical significance of the output spectrum is the rate limiting step. It scales roughly linearly with the number of iterations used to build the distribution. For reference, 1,000 iterations require approximately one day to run and 10,000 require over 10 days (Lines 535-537). Assessing the relationship between length of data and the runtime of a single iteration is complicated by the generic solver, it’s initial estimate, its rate of convergence to the global minimum (factors we did not control), and the choice of subject-specific delta (or error) term. Therefore, we elected not to derive an expression relating the length of the input data to the runtime. We hope that the runtimes described above sufficiently illustrate practical realities of implementing our method.***

2. One major concern is that the peaks of BPWP shown in Figure 3F and 4F do not appear to align well with the CWT results, which exhibit only a few modes. While the paper states that the spectrum aligns visually with the main peaks in some examples and the methods section discusses quantitative comparisons between the two, I could not find these more quantitative results.

***The recommendation for a more explicitly quantitative comparison would absolutely strengthen this presentation. Comparing the sparse, narrowband output spectra of BPWP with the dense, continuous wavelet transform outputs in both the frequency and time domain is a nontrivial task. This consideration, together with the hypothesized frequency-dependence of model performance, prompted us to evaluate model performance in a frequency-specific manner. Briefly, we bandpass filtered the original signal and the model output with central periods ranging from 1-120 days (Methods: lines 517-531), then calculated the Pearson correlation coefficient between the two filtered timeseries for each band. The results are now depicted in Figure 7, Supplementary figures 10 and 11, and discussed in the results (Lines 269-287) and discussion (Lines 317-326). Method performance as captured in correlation coefficients was indeed frequency dependent, with highest correlations for the longer cycles above 20-30 days. This indicates that model performance is best for longer cycles, as one would expect given the anticipated increased uncertainty at higher frequencies when sampling at a rate of only 5 random samples per day as we are in this implementation of BPWP. Notably, correlation coefficients for the bands evaluated were very high, largely above 0.8 and approaching 1, indicating very close agreement between the cycles in the original data and those estimated by BPWP.***

3. Figure 2 is not referenced in the main text. It is essential to include appropriate references to all figures within the text to guide readers and ensure proper understanding of the paper's content.

***Figure 2 is now referenced in the results (Line 157) in addition to the methods.***

4. The mention of "figures 3 and 4" should be capitalized, as they represent figure titles.

***This has been corrected (Line 214).***

5. In the discussion of the limitations of Classical Fourier Transform, the statement that it requires dense and regular sampling should be clarified. While Fast Fourier Transforms may have such requirements, the Fourier Transform itself is not inherently limited to regular sampling. It would be more accurate to state that the computation of sines and cosines for the Fourier Transform can be computationally intensive, making it less suitable for sparse time series.

***The reviewer’s concerns about the use of more precise language to describe limitations of different implementations of the Fourier Transform are well taken. We clarified the language in the introduction (Lines 104-105) and added mention of the non-uniform discrete Fourier transform as an alternative, Fourier-based method that accommodates irregular sampling in the discussion (lines 334-340).***

6. Methods Section: The paper briefly mentions the hardware for extracting the Interictal Epileptiform Discharges (IEDs) but lacks a detailed explanation of the actual algorithm used. It is suggested to include a sentence or two describing the method employed, or at least provide a reference to the specific algorithm along with the hardware information. This addition would enhance the clarity of the methods section.

***This recommendation is well received given the complexity associated with and variability in spike and IED detectors. The methods section on IEDs now includes an additional summary of the IED detector from Janca et al. that we used (Lines 388-391).***
